# Identification and Validation of Four Serum Biomarkers With Optimal Diagnostic and Prognostic Potential for Gastric Cancer Based on Machine Learning Algorithms

**DOI:** 10.1002/cam4.70659

**Published:** 2025-03-14

**Authors:** Yi Liu, Bingxian Bian, Shiyu Chen, Bingqian Zhou, Peng Zhang, Lisong Shen, Hui Chen

**Affiliations:** ^1^ Department of Clinical Laboratory Xinhua Hospital, Shanghai Jiao Tong University School of Medicine Shanghai China; ^2^ Institute of Artificial Intelligence Medicine, Shanghai Academy of Experimental Medicine Shanghai China; ^3^ Faculty of Medical Laboratory Science, College of Health Science and Technology Shanghai Jiao Tong University School of Medicine Shanghai China

**Keywords:** diagnostic and prognostic potential, gastric cancer, integrated bioinformatics analysis, promising biomarkers, RT‐PCR and ELISA

## Abstract

**Background:**

Gastric cancer (GC) is considered a highly heterogeneous disease, and currently, a comprehensive approach encompassing molecular data from various biological levels is lacking.

**Methods:**

This study conducted different analyses, including the identification of differentially expressed genes (DEGs), weighted correlation networks (WGCNA), single‐cell RNA sequencing (scRNA‐seq), mRNA expression‐based stemness index (mRNAsi), and multiCox analysis, utilizing data from Gene Expression Omnibus (GEO) and The Cancer Genome Atlas (TCGA) databases. Subsequently, the machine learning algorithms including least absolute shrinkage and selection operator (LASSO) regression and random forest (RF), combined with multiCox analysis were exploited to identify hub genes. These findings were then validated through the receiver operating characteristic (ROC) curve and Kaplan–Meier analysis, and were experimentally confirmed in GC samples by reverse transcription–polymerase chain reaction (RT‐PCR) and enzyme‐linked immunosorbent assay (ELISA).

**Results:**

Integrated analysis of TCGA and GEO databases, coupled with LASSO regression and RF algorithms, allowed us to identify 18 hub genes encoding differentially expressed secreted proteins in GC. The results of RT‐PCR and bioinformatics analysis revealed four promising biomarkers with optimal diagnostic and prognostic potential. ROC analysis and Kaplan–Meier curves highlighted CHI3L1, FCGBP, VSIG2, and TFF2 as promising biomarkers for GC, offering superior modeling accuracy. These findings were further confirmed by RT‐PCR and ELISA, affirming the clinical utility of these four biomarkers. Additionally, CIBERSORT analysis indicated a potential correlation between the four biomarkers and the infiltration of B memory cells and Treg cells.

**Conclusion:**

This study unveiled four promising biomarkers present in the serum of patients with GC, which could serve as powerful indicators of GC and provide valuable insights for further research into GC pathogenesis.

**Abbreviations**
ANKRD1: ankyrin repeat domain 1
Ap‐1: activator protein‐1
AUC: area under the curve
BP: biological pathways
C2: complement component 2
CC: cellular components
CG: chronic gastritis
DEGs: differentially expressed genes
EBV: Epstein–Barr virus
FDR: false discovery rate
GC: gastric cancer
GEO: Gene Expression Omnibus
GO: Gene Ontology
GS: gene significance
HCC: hepatocellular carcinoma
HER2: human epidermal growth factor receptor 2
HPA: Human Protein Atlas
KDMs: histone lysine demethylases
KEGG: Kyoto Encyclopedia of Genes and Genomes
LogFC: log fold change
MF: molecular functions
MM: module membership
MRGs: mitophagy‐related genes
mRNAsi: mRNA expression‐based stemness index
MSI: microsatellite instability
multiCox: multivariate Cox
NMF: nonnegative matrix factorization
OS: overall survival
PCA: principal component analysis
PCs: principal components
PD‐L1: programmed cell death ligand 1
PPI: protein–protein interaction
QC: quality control
ROC: receiver operating characteristic curve
scRNA‐seq: single‐cell RNA sequencing
SNP: single nucleotide polymorphism
TCGA: The Cancer Genome Atlas
TCGA‐STAD: TCGA stomach adenocarcinoma
t‐SNE: t‐distributed stochastic neighbor embedding algorithm
WGCNA: weighted correlation network analysis

## 1 Background

Gastric cancer (GC) currently ranks fourth among the most prevalent malignancies worldwide and is the second leading cause of cancer‐related deaths [1, 2]. Its highest incidence and mortality rates are observed in East Asia, specifically in Korea, Mongolia, Japan, and China [3]. *Helicobacter pylori* infection is the primary risk factor for GC, although genetic and environmental factors also contribute significantly [4]. Surgical resection combined with chemotherapy remains the primary treatment option for GC [5, 6]. However, despite advances in our understanding of GC at the molecular and cellular levels, therapeutic options remain limited, offering only modest survival benefits for most patients [7]. Consequently, there is an urgent need to identify novel therapeutic targets and reliable biomarkers for GC prediction, driven by a large number of studies.

The biomarkers playing a pivotal role in GC prediction have been suggested by a large of evidence. Traditional biomarkers such as CEA (carcinoembryonic antigen) and CA19‐9 (carbohydrate antigen 19‐9) exhibit limited sensitivity, while the significance of HER2 in prognosis prediction remains inconclusive [8, 9]. Hence, there is a compelling need to develop new, sensitive, and specific biomarkers to cater to the demands of clinical precision medicine. Bioinformatics analysis has emerged as the mainstream approach for identifying and exploring cancer‐related biomarkers associated with uncontrolled cellular growth and metastasis [10, 11]. Over the past few decades, several researchers have tried to explore disease‐related genes in GC, including key genes [12, 13], diagnostic markers [14]^,^ and prognostic indicators [15]. While some disease‐related genes have been identified in GC, the quest for efficient and specific biomarker candidates with transformative clinical potential continues.

The present study performed an integrated bioinformatic analysis to identify four promising biomarkers for GC. These biomarkers were discovered through various bioinformatic analyses, and a diagnostic and prognostic model for diagnosis and prognosis prediction was constructed using least absolute shrinkage and selection operator (LASSO) regression and random forest (RF) algorithms. These biomarkers were thoroughly validated through reverse transcription–polymerase chain reaction (RT‐PCR) and enzyme‐linked immunosorbent assay (ELISA) experiments, demonstrating their strong predictive performance. Based on the results, these four biomarkers can be considered to hold great potential for future experimental and clinical studies in the context of GC.

## 2 Materials and Methods

### 2.1 Data Collection and Processing

The transcriptomic and clinical characteristic data of GC samples were downloaded from The Cancer Genome Atlas (TCGA) and Gene Expression Omnibus (GEO) databases. The GC mRNA profile datasets, including GSE2669, GSE191275, GSE163416, GSE130823, GSE55696, and GSE108602, and the single‐cell RNA sequencing (scRNA‐seq) dataset from GSE11230 were downloaded. For TCGA Stomach Adenocarcinoma (TCGA‐STAD) dataset, the Ensembl IDs of the 375 GC and 32 normal samples were converted into gene names; the relevant clinical data were matched to each sample and all of them were merged using Perl language.

### 2.2 Differentially Expressed Genes (DEGs) Analysis

The software R (version 3.6.0) and related packages including limma package and pheatmap were used to screen DEGs, and the significant difference was defined by Wilcoxon test between control and tumor groups. The log fold change referred as LogFC should be > 1 and false discovery rate referred to as FDR should be < 0.05, which were set to narrow down the screening range.

### 2.3 scRNA‐Seq Analysis

The package of Seurat R was used to determine the quality control (QC), and the function of Percentage Feature Set was employed to compute the percentage of mitochondrial genes [16]. Following normalizing data with the Seurat “Normalize Data” function, we confirmed the highly variable genes including 1500 genes. The t‐distributed stochastic neighbor embedding (t‐SNE) algorithm was performed to downscale the principal components (PCs). After that, nine clusters were obtained and the “JackStraw” was drawn based on cluster differentiation [17]. The LogFC > 0.5 and FDR < 0.05 were the thresholds to determine the marker genes in each cluster, and the top 10 in each cluster were displayed on heatmap. By using “SingleR” package, each cluster was matched, defined, and identified based on cellular markers [18]. And then the R package “Monocle2” was used, and the trajectory diagram was drawn to reveal the cell cluster differentiation [19].

### 2.4 mRNA Expression‐Based Stemness Index (mRNAsi) Analysis and Construction of Weighted Correlation Networks (WGCNA)

The mRNAsi was calculated based on the molecular spectrum of cell stemness [20]. Clinical data of each GC sample obtained from TCGA were matched with the calculated mRNAsi. Kruskal–Wallis test was used to determine the significance of differences between normal and tumor samples. Then, the overall survival (OS) analysis, the difference between normal and tumor samples, and the changes in pathological grades and staging were analyzed according to mRNAsi scores with unpaired *t*‐test.

For WGCNA, the similarity matrix was constructed based on the soft‐thresholding power β calculated by R package. Gene significance (GS) refers to the correlation between genes and sample traits. The coexpression similarity and clinical phenotypes were incorporated and defined as the different gene modules. Module membership (MM) was defined as the correlation between the genes within the module and the gene expression profile. After that, genes within the GS and MM were analyzed based on the selected module.

### 2.5 Identification of Hub Genes

After integrating all kinds of analytic results, we used the Lasso regression and RF algorithms to predict candidate genes in GC with the “glmnet” package. Finally, the multiCox analysis was used to dig out 18 hub genes and further analysis was performed. The multiCox regression could divide GC patients into two groups based on the median risk score. The Kaplan–Meier (K‐M) analysis was established to obtain the survival time of the high‐risk and low‐risk groups. The receiver operating characteristic (ROC) was analyzed to compare the specificity and sensitivity of model predictions.

### 2.6 Prognostic Risk Analysis

The clinical data of GC samples from TCGA‐STAD including the corresponding age, gender, TNM (tumor node metastasis) stage, AJCC (American Joint Committee on Cancer) stage were downloaded, and the survival status of GC samples was incorporated. The model's capabilities were analyzed by the “survival” package in R software. Meanwhile, we integrated the prognostic signatures and clinicopathological characteristics to construct a nomogram to visually evaluate survival rate of the patients.

### 2.7 Analysis of Gene Function

Gene functional analysis was applied to verify the potential functions of hub genes with the “clusterProfiler” R package. For Gene Ontology (GO) analysis, the molecular functions (MF), biological pathways (BP), and cellular components (CC) were analyzed and visualized by R software. KEGG analysis was conducted to identify the related pathways of hub genes. Adjust *p* value (Adj.*p*) < 0.05 was considered statistically significant.

### 2.8 Protein–Protein Interaction (PPI) Network Construction

To explore the interaction among hub genes, the 18 hub genes were imported into the STRING. The PPI of hub genes was constructed using STRING.

### 2.9 Immune Infiltration Analysis

The immune landscape of the tumor microenvironment (TME) was assessed, and the level of immune cell infiltration was quantified by the R ESTIMATE package [21]. GC patients were categorized into the CHI3L1^low^ + FCGBP^high^ + VISIG2^high^ + TFF2^high^ (*n* = 35) and the CHI3L1^high^ + FCGBP^low^ + VISIG2^low^ + TFF2^low^ group (*n* = 16) based on the median expression levels of CHI3L1, FCGBP, VSIG2, and TFF2. The distributions of immune cells between the CHI3L1^high^ + FCGBP^low^ + VSIG2^low^ + TFF2^low^ and CHI3L1^low^ + FCGBP^high^ + VSIG2^high^ + TFF2^high^ group were compared using the CIBERSORT algorithm [16]. The ggplot2 R package was used to show the distribution of immune cell infiltrations in TCGA dataset.

### 2.10 RNA Extraction and Quantitative Real‐Time PCR

Total RNA was extracted with TRIzol reagent (Invitrogen, USA) according to the manufacturer's instructions. Following the concentration and quality of the total RNA determined by NanoDrop spectrophotometer (Thermo Fisher Scientific, USA), the reverse transcription was performed using PrimeScript RT master mix (TaKaRa, Japan). Quantitative real‐time PCR (qRT‐PCR) was performed on the 7900 HT real‐time PCR system (Applied Biosystems, USA) using SYBR Premix Ex Taq (TaKaRa, Japan). The expression level of actin was used as an endogenous control. The primers used for the experiments are listed in Table S1.

### 2.11 ELISA Analysis

Serum CHI3L1, FCGBP, VSIG2, and TFF2 levels were detected by ELISA assay. In short, we first transferred 100 μL serum from each sample to a 96‐well plate containing the anti‐CHI3L1, anti‐FCGBP, anti‐VSIG2, and anti‐TFF2 antibodies. Following mixing with antibodies, the wells were incubated and washed, and the protein levels were calculated based on the standard curve.

### 2.12 Human GC Samples

The tissues and serum of GC were obtained from the Xinhua Hospital, Shanghai Jiao Tong University School of Medicine, between 2009 and 2023 (Tables S2–S4), and then stored at −80°C. When needed, the samples were subjected to mRNA and protein extraction. All patients were diagnosed by pathological analyses based on the TNM criteria defined by the International Union Against Cancer (UICC). The protocol in this study conformed to the ethical guidelines of the Declaration of Helsinki and was approved by the Institutional Review Board and Ethics Committee of Xinhua Hospital.

### 2.13 Cells and Cell Culture

The GC cell line (HGC‐27) was purchased from the Cell Bank of the Chinese Academy of Sciences (Shanghai, China). Cells were cultured at 37°C in an atmosphere containing 5% CO_2_ in Dulbecco's modified Eagle's medium (DMEM, HyClone, USA). Besides, 10% fetal bovine serum and 1% penicillin/streptomycin (Gibco, USA) were supplemented into the medium.

### 2.14 Small Interfering RNAs (siRNAs) Production and Transfection

siRNAs of four genes including CHI3L1, FCGBP, VSIG2, and TFF2 were designed and synthesized by Hippo Biotec (HuZhou, China). SiRNAs were mixed with Lipofectamine 3000 (Invitrogen, USA) for 5 min in Opti‐MEM (Gibco, USA), and then transferred into the medium containing 10% FBS. Cells were collected after 48 h and used to perform the subsequent experiments.

### 2.15 Cell Viability Assay

A total of 3000 cells per well of HGC‐27 cells were counted and seeded in a 96‐well plate for 1–5 days, and cell viability was detected with Cell Counting Kit‐8 (CCK8) (Dojindo Laboratories, Japan). The optical density at 450 nm was measured to determine the cell viability at 24 h intervals.

### 2.16 Cell Apoptosis With Flow Cytometry (FCM) Analysis

The Apoptosis Detection Kit I (BD Pharmagen, USA) was used to analyze the cell apoptosis, as previously described [22].

### 2.17 Statistical Analysis

The *t*‐test/variance and chi‐square test were used to analyze the distributions of dichotomous variables, respectively. The log‐rank test and K‐M statistics were performed to compare the survival rate. The software including Perl and R were adopted for bioinformatic analysis, and the statistical significance was defined as *p* < 0.05.

## 3 Results

### 3.1 Acquisition of DEGs

**FIGURE 1:**
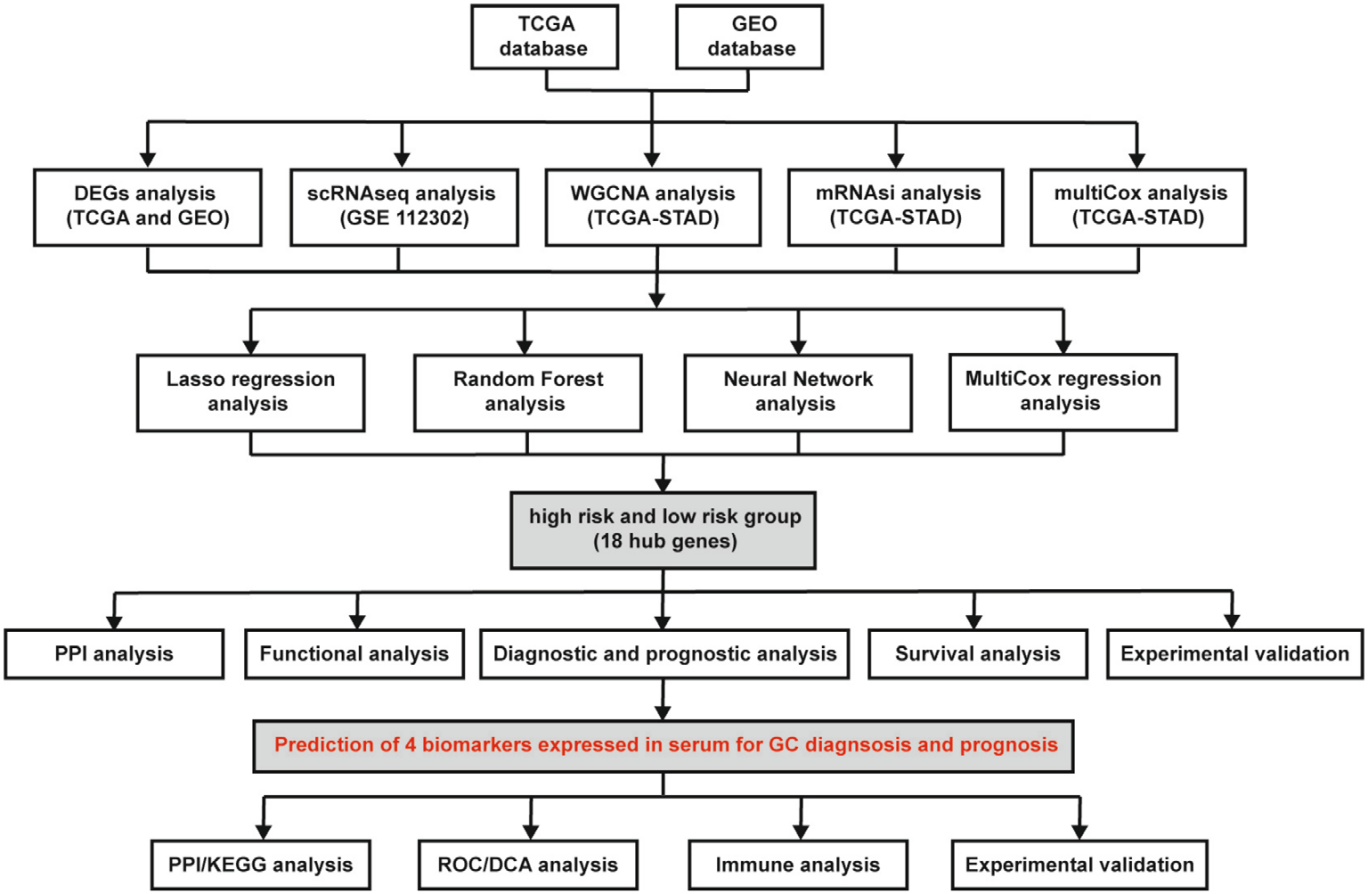
The workflow of the study.

This study integrated TCGA and GEO datasets and conducted a systematic analysis of DEGs in GC. Figure 1 illustrates the entire research process. The results of an integrated analysis of scRNA‐seq and WGCNA/mRNAsi from GEO (GSE 112302) and TCGA databases revealed 389 candidate genes associated with GC development. Further analyses involved comparisons between patients with GC and those with gastritis (351 genes) and healthy individuals (4086 genes). Subsequently, 18 hub genes were identified, which were further verified by clinical and experimental validation using RT‐PCR assay. Finally, RT‐PCR and ELISA assays were conducted to verify the expression of the four biomarkers in GC tissues and serum samples.

### 3.2 Identification of Stem Cell‐Related Genes in GC

Stem cells, characterized by self‐renewal and therapeutic resistance characteristics, play pivotal roles in metastasis, drug resistance, and GC recurrence. This study identified key genes associated with stemness by combining the scRNA‐seq dataset from GSE112302 and mRNAsi data from TCGA.

The scRNA‐seq analysis involved 10 samples from six GC and four normal tissues. To reduce dimensionality, the R package was employed for QC and PC analyses (Figure S1A,B). Additionally, the top 20 PCs were determined using the JackStraw procedure (Figure S1C). The results revealed nine clusters through t‐SNE analysis (Figure S1D) and identified DEGs as the marker genes. The top 10 genes from various clusters were displayed in a heatmap (Figure S1E). Pseudo‐time and trajectory analyses indicated that Clusters 0, 1, 2, 3, 4, 5, and 8 were defined as epithelial cells, Cluster 6 as fibroblasts, and Cluster 7 as macrophages (Figure S1F). Complementing these findings with GO analysis, the findings led to the speculation that Clusters 3 and 4 may be related to stem cells, which resulted in the selection of 1943 marker genes for further analysis (Figure S1G).

mRNAsi serves as an index quantifying the correlation between tumor cells and stem cells, representing cancer cell stemness [23]. GC tissues scored higher than normal tissues as shown in Figure S2A. Intricately, patients with GC who have higher mRNAsi scores exhibited better OS than those with lower mRNAsi scores (Figure S2B). Additionally, patients who had G2 grade had the highest mRNAsi scores, likely due to the relatively small sample size of patients who had G1 grade (Figure S2C). Notably, significant differences in mRNAsi scores were observed between Stage I and other stages (Figure S2C). Subsequently, WGCNA was used to explore genes associated with GC stemness and identified the brown and blue modules as having a higher correlation with mRNAsi (Figure S2D). A total of 941 and 903 genes were identified from scRNA‐seq and mRNAsi analyses, respectively, with 80 overlapping genes (Figure S2E). A union set comprising 1764 stem cell–related genes was incorporated for further analysis, referred to as “stem.”

### 3.3 Identification of Hub Genes Based on Multiple Steps of Screening

**FIGURE 2:**
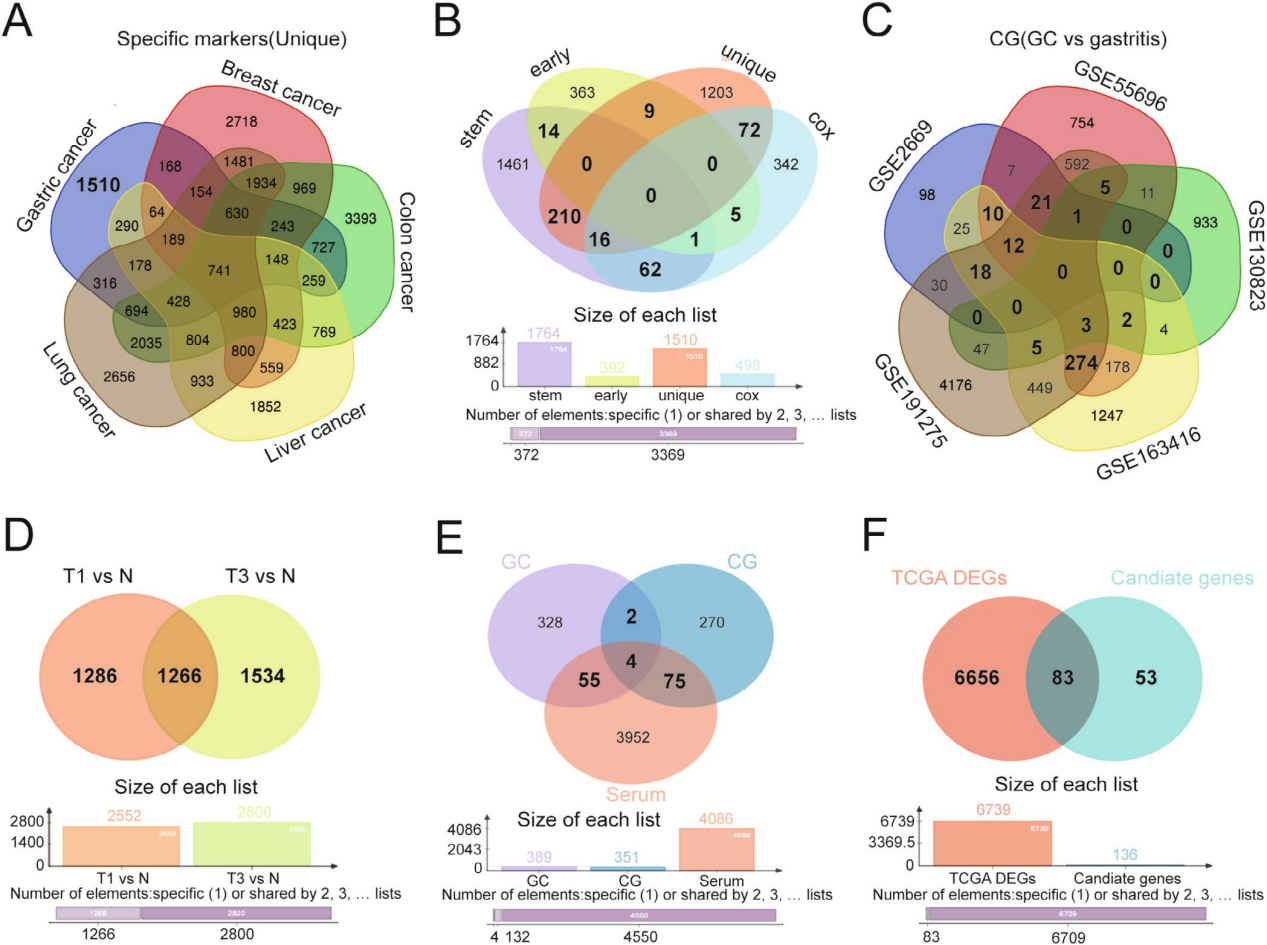
Identification of hub genes based on multiple steps of screening. (A) Venn diagram of the specific markers expressed in GC compared with those expressed in breast cancer, colon cancer, liver cancer, and lung cancer. (B) The common differential analysis of stem cell‐related genes, early expression genes, specific genes, and genes analyzed by multiCox regression. (C) Differential analysis of DEGs in GC is different from CG. (D) The common DEGs in serum of T1 vs. N and T3 vs. N. T1 vs. N: Early carcinoma patients versus normal, T3 vs. N: T3 advanced carcinoma patients versus normal. (E) Venn plot of the overlapping genes identified through results of three analyses including DEGs from GC vs. healthy people, GC vs. CG, and from serum of GC patient vs. healthy people. (F) Overlapping DEGs from TCGA and candidate genes resulted from above analyses.

To identify specific markers expressed in GC, the DEGs in GC were compared with four other cancers, including breast cancer, colon cancer, liver cancer, and lung cancer. A total of 1510 genes expressed only in GC were identified for downstream analyses, referred to as “unique” (Figure 2A). Subsequent multiCox analyses (“cox”) and GC early expression gene analysis (“early”) were performed. Combining the results of these four analyses, 389 genes were identified as candidates in more than two analyses, suggesting their involvement in driving GC development and progression (Figure 2B). Furthermore, five GEO datasets were screened to identify DEGs between patients with GC and patients with chronic gastritis (CG), identifying 351 genes present in more than three GEO datasets (Figure 2C). Additionally, 4086 DEGs were identified in serum of T1 and T3 patients compared with healthy individuals in the GSE108602 dataset (Figure 2D). Finally, by integrating the results from these three analyses, including DEGs from GC versus healthy individuals, GC versus CG, and serum of patients with GC versus healthy individuals, 136 candidate genes were selected for further analysis (Figure 2E). After overlapping these genes with those from the TCGA database and the previous analyses, 83 GC‐related genes were identified for final analysis (Figure 2F).

### 3.4 Establishment of Hub Genes and Functional Analysis

**FIGURE 3:**
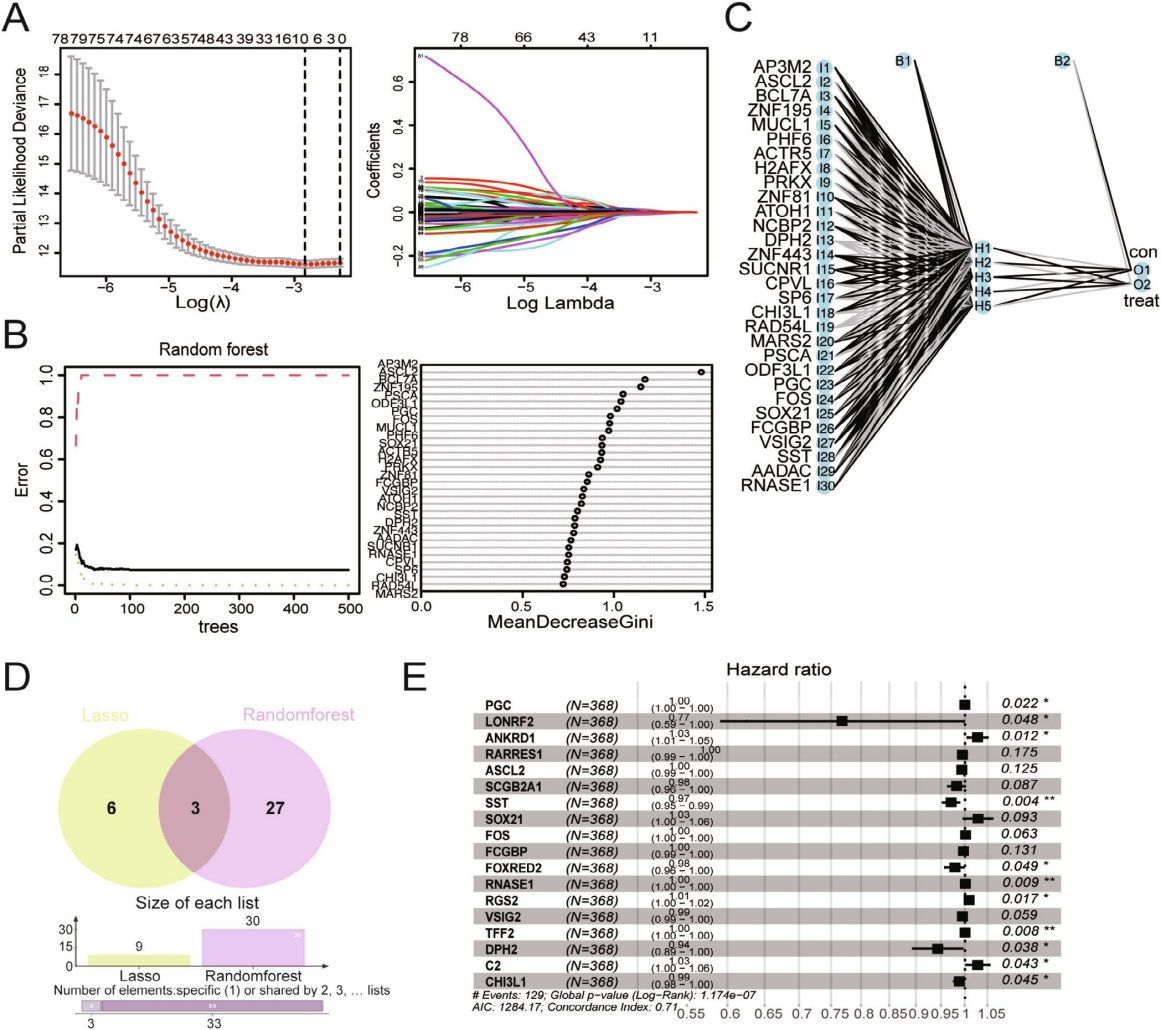
Establishment of 18 hub genes using machine learning. (A) Lasso regression analysis and the number of variables corresponding to the optimal λ value. (B) Screen biomarkers based on Random Forest algorithm. (C) Plot diagram of highest predictive neural net function. (D) Venn diagram of the intersection of the diagnostic markers obtained from two algorithms. (E) MultiCox stepwise regression analysis of the above results to identify the hub genes.

To establish an ideal model for diagnosing and evaluating the prognosis of GC, two machine learning algorithms were used, including LASSO regression and RF, to identify feature genes in GC. After eliminating collinearity through LASSO regression, nine genes were selected from the statistically significant univariate variables (Figure 3A). Subsequently, RF analysis was conducted in conjunction with feature selection, and the results revealed that 30 genes exhibited relative correlation in descending order (Figure 3B). Additionally, neural network analysis was performed to assess the risk prediction associated with these 30 genes (Figure 3C). A Venn diagram analysis showed that only three genes overlapped with the previous results (Figure 3D). To gather more information for subsequent analysis, a union set comprising 36 genes was selected for multiCox regression analysis. As shown in Figure 3E, 18 genes were recognized as hub genes related to GC prognosis, including PGC, LONRF2, ANKRD1, RARRES1, ASCL2, SCGB2A1, SST, SOX21, FOS, FCGBP, FOXRED2, RANSE1, RGS2, VSIG2, TFF2, DPH2, C2, and CHI3L1. However, only 11 genes of them had significant *P* values, with their levels being associated with the OS of patients with GC, including PGC, LONRF2, ANKRD1, SST, FOXRED2, RANSE1, RGS2, TFF2, DPH2, C2, and CHI3L1. Although there was no significant difference among the other seven genes, they were also considered to play important roles in the model.

The STRING was used to reveal the interaction network of these hub genes to explore their relationships at the protein levels. As shown in Figure S3A, FOS exhibited associations with SST and RGS2, whereas PGC demonstrated a significant interaction with TFF2. This study subsequently verified the interactions among these genes based on their expression in the TCGA database (Figure S3B). To further analyze the functions of the 18 hub genes, the GO and KEGG analyses were performed (Figure S3C).

### 3.5 Diagnostic and Prognostic Analysis of the Hub Genes

**FIGURE 4:**
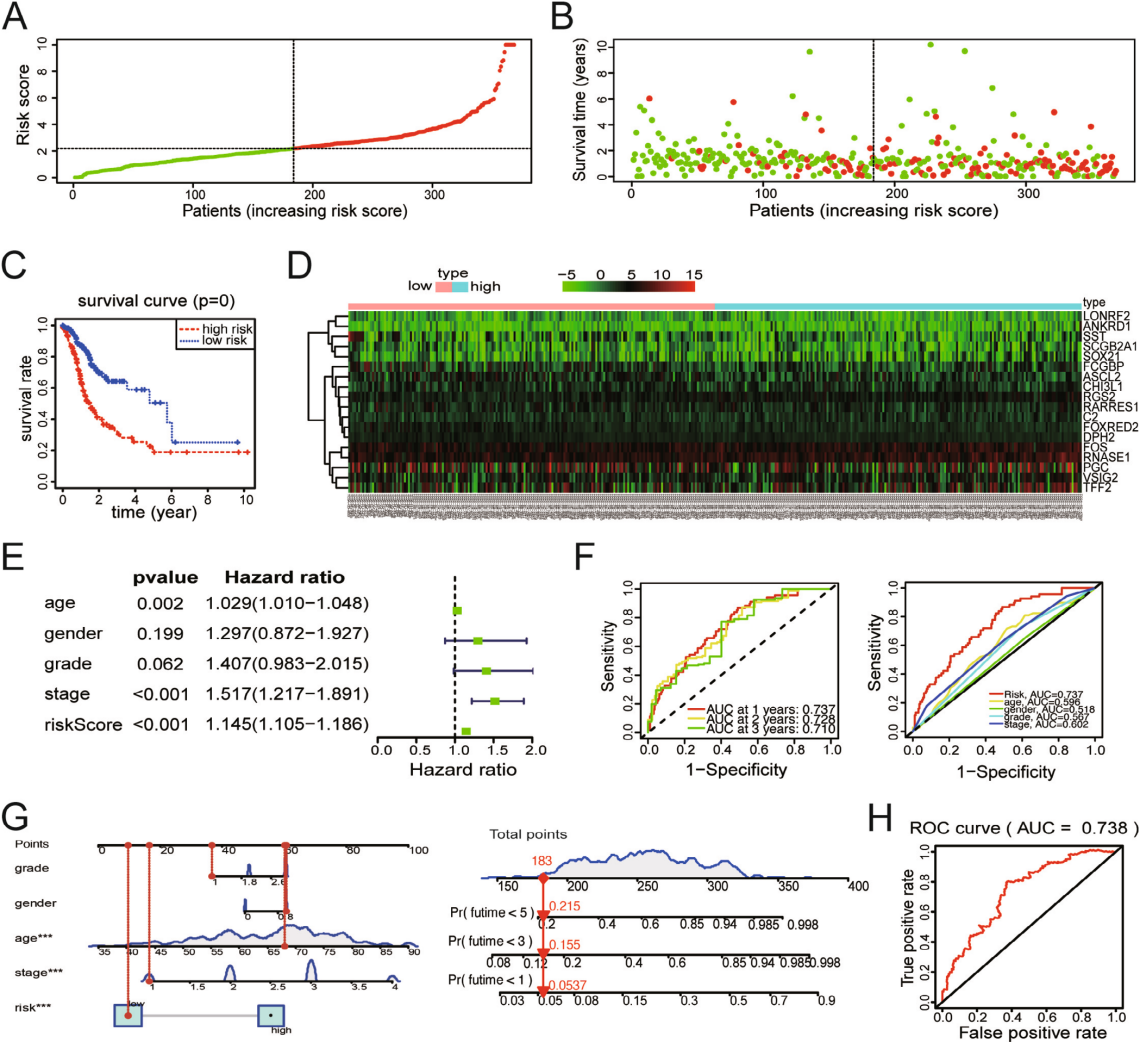
Verification of the diagnostic and prognostic value of hub genes. (A) Distribution of risk scores per patient. (B) Relationships between OS and survival time of GC patients ranked based on risk score. (C) Kapan–Meier analysis of OS of the high‐risk and the low‐risk groups. (D) Heatmap representing the expression profiles of the 18 hub genes. (E) Forest plot depicting the associations between risk factors and other clinical features and the prognosis of GC patients. (F) ROC analysis for OS prediction according to the survival time and other clinical features. (G) The nomogram of the model predicting the occurrence of GC. (H) The ROC of the diagnostic efficacy verification.

To assess the clinical application value of the 18 hub genes, GC patients obtained from TCGA database were categorized into high‐risk and low‐risk groups based on the media risk score derived from multiCox regression. Dot‐plot visualizations were used to display corresponding signatures, including risk score and survival status (Figure 4A,B). Based on the results of K‐M analysis, OS in the high‐risk group was shorter than in the low‐risk group (*p* < 0.001) (Figure 4C). Further examination of gene expression in both two groups found that some of them were highly expressed in the high‐risk patients (Figure 4D). MultiCox regression analysis revealed the different prognostic factors for GC, including age, stage, and risk score (Figure 4E). Time‐dependent ROC curve analysis, as shown in Figure 4F, revealed that all 18 hub genes served as excellent indicators for prognosis prediction within 3 years. In addition, an area under the curve (AUC) analysis demonstrated that risk score provided the best indicator for prognosis prediction among age, gender, grade, and stage (Figure 4F). A nomogram based on the risk score of 18 genes and clinical characteristics was constructed to predict the OS of GC patients (Figure 4G). Moreover, the ROC curve analysis suggested the diagnostic efficiency of the 18 hub genes, yielding an AUC value of 0.738 (Figure 4H). Therefore, these 18 hub genes may hold clinical utility for managing patients with GC.

### 3.6 Expression and OS Analysis of the Hub Genes

After building a model containing the 18 hub genes described above, we examined differential mRNA levels between GC and normal tissues in the TCGA‐STAD database (Figure S4A). Eleven of 18 hub genes were differentially expressed in the GC versus CG groups, 5 were differentially expressed in the GC versus N groups and 2 were coexpressed in both groups (Figure S4B,C). Notably, all of them could be detected in serum of GC patients. Except for PGC, the expression trends of the 17 genes in T1 versus N and T3 versus N were consistent (Figure S4D). Furthermore, this study examined the levels and cellular localization of the proteins encoded by these genes using clinical samples from the Human Protein Atlas (HPA) database (Figure S4E,F). PCR assay results verified the expression of the 18 hub genes, and significant differences in the expression of 12 genes were observed in 10 GC tissues and matched control tissues (Figure S5). Moreover, K‐M analysis revealed that high expression of ANKRD1, ASCL2, CHI3L1, DPH2, C2, FCGBP, FOXRED2, RGS2, and TFF2 in GC was associated with shorter OS, which was consistent with their high expression in GC (Figure S6).

### 3.7 Prediction and Validation of Four Promising Biomarkers in Serum of GC

**FIGURE 5:**
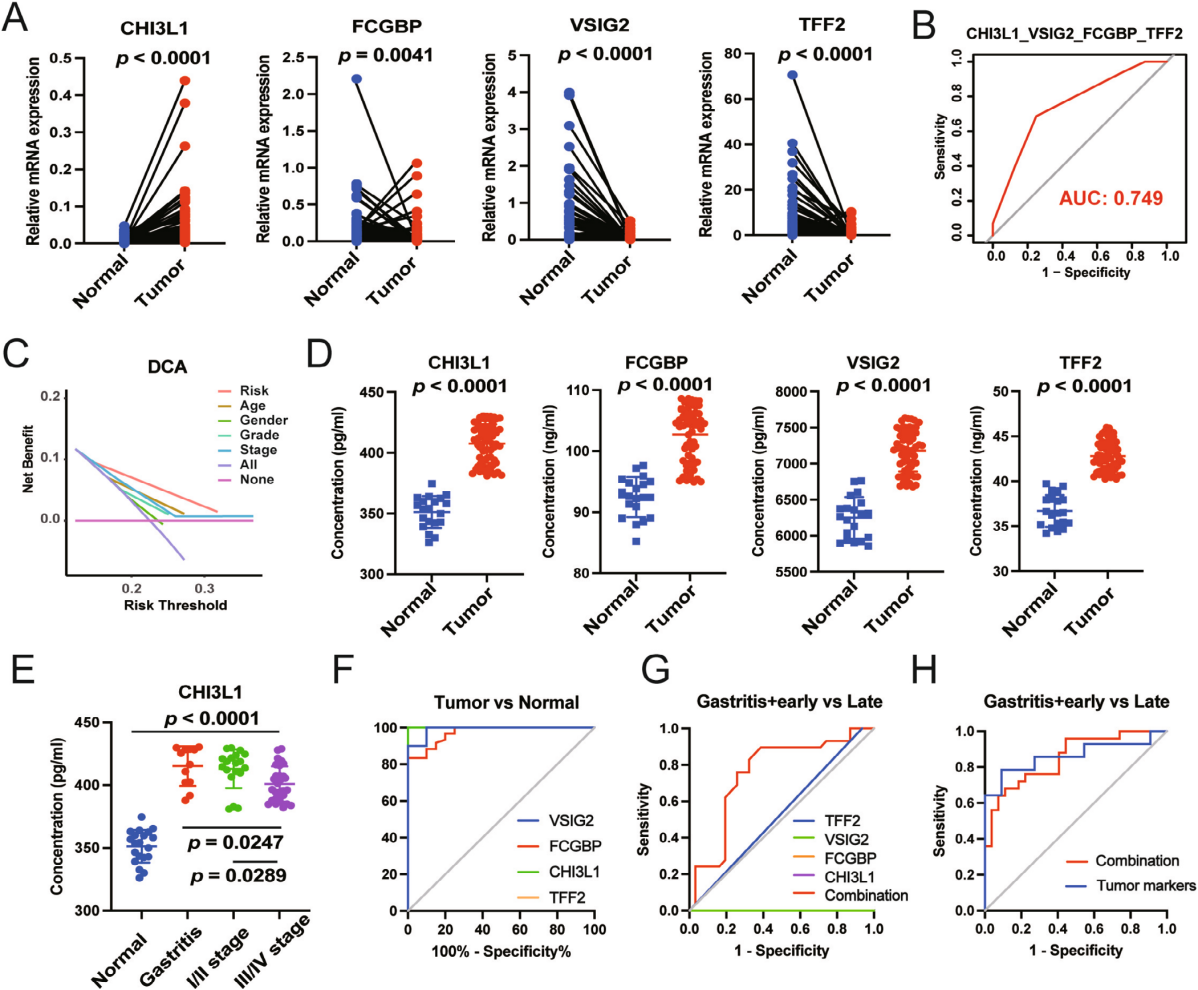
Identification of four promising biomarkers in serum of GC. (A) qRT‐PCR analysis of the expressions of CHI3L1, FCGBP, VSIG2, and TFF2 in 54 pairs of normal and GC tissues. (B) ROC analysis of the combination of CHI3L1, FCGBP, VSIG2, and TFF2 for GC prediction based on TCGA database. (C) DCA analysis for the prognostic prediction according to risk factors and other clinical features. (D) ELISA analysis of the protein levels of CHI3L1, FCGBP, VSIG2, and TFF2 in serum of 20 normal samples and 60 GC patients. (E) The protein level of CHI3L1 in serum of 20 normal samples, 12 gastritis patients, 19 I/II stage of GC patients, and 29 III/IV stage of GC patients. (F) ROC analysis for the diagnosis of GC based on the four biomarkers expressed in serum. (G) ROC analysis for evaluating the diagnostic efficiency of distinguishing gastritis patients + early‐stage GC patients from late‐stage GC patients with the single biomarker and the combining four biomarkers. (H) ROC analysis of the combining 4 biomarkers and 11 tumor markers (AFP, CEA, CA199, CA724, CA125, CYFRA211, CA153, CA242, NSE, SCC, and CA50) in distinguishing gastritis patients + early‐stage GC patients from late‐stage GC patients.

To further optimize the diagnostic accuracy of this model, RT‐PCR combined with stepwise ROC curve analyses were performed. As shown in Figures 5A and S7A, the mRNA levels of CHI3L1 and FOXRED2 were significantly upregulated in 54 GC samples compared with normal tissues. However, with the different pattern, the mRNA levels of FCGBP, VSIG2, TFF2, SCGB2A1, and SST appeared to be less expressed in GC patients. Subsequently, PPI and KEGG analyses were conducted, and the results revealed an interaction pattern between FCGBP2 and TFF2, as well as the enrichment of the gastric acid secretion pathway (Figure S7B,C). Furthermore, stepwise ROC curve analysis revealed that the combination of CHI3L1, FCGBP, VSIG2, and TFF2 yielded the highest AUC value (Figures 5B and S7D, Table S4). And the decision curve analysis (DCA) of the above four genes demonstrated that the risk index of patients with GC was an excellent prognostic indicator (Figure 5C). Since all four biomarkers were secretary, their expressions were further compared in serum of normal and GC samples. As shown in Figure 5D, CHI3L1 exhibited higher expression in the serum of patients with GC, consistent with its transcriptional level. However, the protein levels of FCGBP, VSIG2, and TFF2 in serum were inconsistent with their transcriptional levels in tissues, namely, higher expression in serum of GC than in healthy individuals (Figure S7E). Additional results revealed that CHI3L1 might have higher expression in the serum of patients with gastritis and patients with early‐stage GC (Figure 5E). This study also assessed the diagnostic efficiency of these four biomarkers for distinguishing between tumor and normal samples through ROC analysis. The results showed that CHI3L1 had an outstanding diagnostic value with the highest AUC value (100%) (Figure 5F). To further demonstrate the role of CHI3L1, FCGBP, TFF2, and VSIG2 on GC cells, we performed cell biology experiments to determine their effects on the cell viability of GC cell lines. As shown in Figure S8A, the mRNA levels of CHI3L1, FCGBP, TFF2, and VSIG2 were high in HGC‐27 cell line. We then transfected the siRNAs into HGC‐27 cell line and the silencing effects were validated by qRT‐PCR (Figure S8B). After transfecting the siRNAs with the most significant silencing effect, we used CCK8 and cell apoptosis analysis to demonstrate the effects of four biomarkers on cell viability in HGC‐27 cell line (Figure S8C,D). Both of the above results revealed that CHI3L1, FCGBP, and VSIG2 played a vital role in the growth of GC cells, but not TFF2. Of course, further studies are needed to confirm the roles of the four biomarkers on GC. The combination of all four biomarkers exhibited higher diagnostic efficiency than any other individual biomarkers, and when combined with eight transitional tumor markers, they showed similar AUC values as those eight markers alone (Figure 5G,H). Collectively, these findings suggest that the four biomarkers, namely, CHI3L1, FCGBP, VSIG2, and TFF2, hold the potential for diagnosing GC, particularly in cases of gastritis and early‐stage GC.

### 3.8 Immune Infiltration Analysis of Four Biomarkers‐Related Immune Cells Subtypes

**FIGURE 6:**
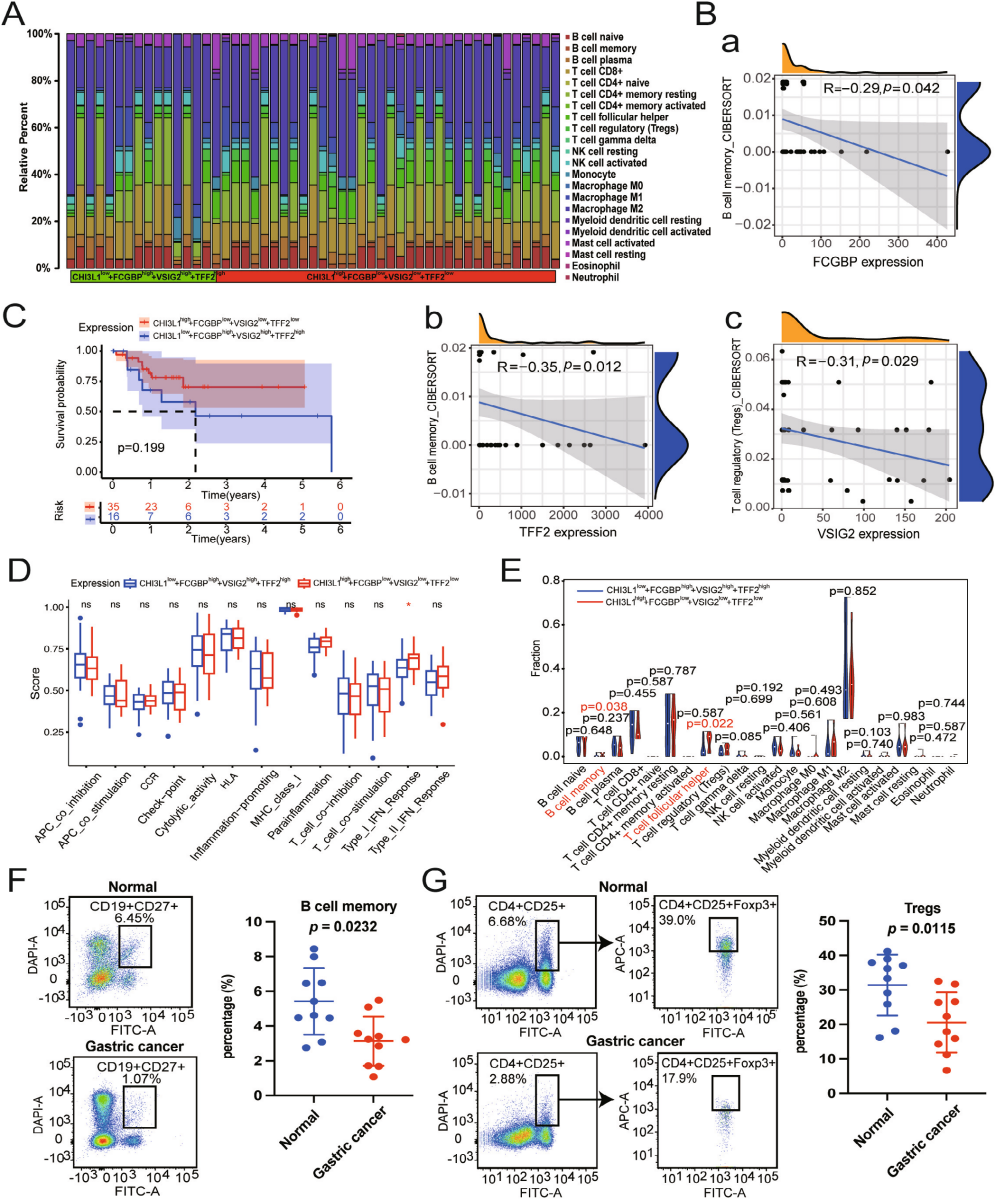
Evaluation of the relationship between four promising biomarkers and immune cells. (A) The proportions of various immune cells in CHI3L1^low^ + FCGBP^high^ + VISIG2^high^ + TFF2^high^ and CHI3L1^high^ + FCGBP^low^ + VISIG2^low^ + TFF2^low^ groups according to the expressions of four promising biomarkers. (B) The correlations between the expressions of FCGBP (A), VSIG2 (B), and TFF2 (C), and the abundance of immune cells in TCGA database. (C) Kaplan–Meier analysis of the effect of the expression of four promising biomarkers on OS of GC patients. (D) The immune function analysis of the two groups in GC. (E) The fraction of immune cells between CHI3L1^low^ + FCGBP^high^ + VISIG2^high^ + TFF2^high^ and CHI3L1^high^ + FCGBP^low^ + VISIG2^low^ + TFF2^low^ groups. (F and G) FCM analysis of B cell memory (F) and Tregs (G) infiltration in the peripheral blood of 10 normal samples and 10 GC patients, and the scatter plots were drawn for statistical analysis.

To assess the effect of the four biomarkers on the overall immune profile of TCGA‐STAD patients, GC patients were categorized into the CHI3L1^low^ + FCGBP^high^ + VISIG2^high^ + TFF2^high^ and CHI3L1^high^ + FCGBP^low^ + VISIG2^low^ + TFF2^low^ group based on the median expression levels of CHI3L1, FCGBP, VSIG2, and TFF2. The distribution of immune cells in both groups was heterogeneous and complex, as shown in Figure 6A. Further analysis revealed a significant negative correlation among FCGBP, TFF2 expression, and B cell memory, as well as VSIG2 expression and T cell regulatory (Tregs) (Figure S9). However, no correlation was observed between CHI3L1 expression and immune cells (Figures 6B and S9). Further investigation of the prognostic values of four biomarkers between the above two groups indicated that the CHI3L1^high^ + FCGBP^low^ + VISIG2^low^ + TFF2^low^ group appeared to have a better prognosis, although the *p*‐value was not significant (Figure 6C). Additionally, the CHI3L1^high^ + FCGBP^low^ + VISIG2^low^ + TFF2^low^ group exhibited characteristics of the TME, with significantly more B cell memory and T cell follicular helper (Tfh) involvement in the Type I IFN response pathway (Figure 6D,E). In conjunction with the correlation and immune cell subtype analyses, the CHI3L1^high^ + FCGBP^low^ + VISIG2^low^ + TFF2^low^ group possessed the higher tumor purity and triggered more immune cell infiltration. The increased infiltration of B cell memory and Tregs in the CHI3L1^high^ + FCGBP^low^ + VISIG2^low^ + TFF2^low^ group might result in lower expression of FCGBP, VSIG2, and TFF2 in GC tissues. On the contrary, the higher expression of FCGBP, VSIG2, and TFF2 in serum might result from the lower B cell memory and Tregs infiltration. To confirm this hypothesis, the percentages of B cell memory and Tregs were analyzed and compared in peripheral blood. FCM analysis revealed the lower B cell memory and Tregs infiltration in the peripheral blood of GC patients, leading to higher expression of FCGBP, VSIG2, and TFF2 in serum (Figure 6F,G).

## 4 Discussion

GC is generally resulted from molecular alterations that lead to mutations of key genes. Major breakthroughs in molecular biology research have spurred research into cancers with clinical diagnostic and prognostic significance [24]. Over the past few decades, the identification of molecular biomarkers has proven valuable for diagnosing, predicting prognosis, and targeting therapy for GC [25, 26, 27]. Besides, a variety of cancer‐related genes and noncoding RNAs were exploited as the diagnostic and prognostic biomarkers of promising targets for GC [28, 29, 30]. Of course, the discovery of ideal diagnostic and prognostic biomarkers with high sensitivity and specificity is necessary and urgent for advancing precision medicine, which stirring up a big wave of application of machine learning and bioinformatic analysis in molecular biology research. Recent research by Wei Wang et al. utilized the nonnegative matrix factorization (NMF) method to establish a novel panel of 24 histone lysine demethylases (KDMs) and performed consensus molecular subtyping based on the TCGA database, stratifying TCGA‐STAS into three clusters. Among these clusters, low KDM gene‐related risk scores showed more effective responses to immunotherapy and chemotherapy [31]. Additionally, Jiarui Wu et al. integrated scRNA sequencing with bulk RNA‐sequencing analysis to explore gene signatures associated with T‐cell infiltration in GC patients, providing new insights into predicting survival time based on immunoinfiltration levels [32]. By combining scRNA‐seq data and transcriptomics, they identified 18 mitophagy‐related genes (MRGs), and a reliable nomogram model was established based on expressions of above genes and clinicopathological parameters. Subsequently, the model was validated to demonstrate the biomarkers for prognosis and candidate targets for GC [33].

The diagnosis and prognosis of GC exhibit significant variability, necessitating the development of novel biomarkers for personalized treatment plans [34]. Compared with previous studies, this research integrated analysis of scRNA‐seq and WGCNA/mRNAsi from GEO and TCGA databases, systematically investigated GC‐related biomarkers expressed in serum through multiple bioinformatic analyses, and then constructed a diagnostic and prognostic signature containing 18 hub genes using machine learning algorithms. Experimental validation was performed to further refine our research objectives, resulting in the identification of four promising GC biomarkers: CHI3L1, FCGBP, VSIG2, and TFF2. These findings enhance convenience and operability for clinical detection. ROC curve analysis revealed the diagnostic value of this panel and its potential for clinical application. ELISA was conducted to validate their predictive value for GC, and the combined application of some tumor markers and the four biomarkers for early‐stage GC diagnosis in serum were explored. The AUC value of the model consisting of the four genes mentioned above (CHI3L1, FCGBP, VSIG2, and TFF2) outperformed other tumor markers and performed well in predicting the prognosis of GC patients. Among the four biomarkers identified, CHI3L1 and TFF2 have been demonstrated to promote tumorigenesis and be associated with OS of GC. CHI3L1 (non‐enzymatic chitinase‐3 like‐protein‐1) is a kind of the glycoside hydrolase family 18, which is synthesized and secreted by various cell types, including immune‐related cells, fibroblast‐like cells, smooth muscle cells, and tumor cells [35, 36]. The latest study revealed that the protein level of CHI3L1 was elevated in serum and predicted the OS of HCC (hepatocellular carcinoma) [37]. CHI3L1 was upregulated in GC and played a significant role in cell growth and metastasis, which was also validated in our study [38]. Targeting CHI3L1 is a therapeutic approach in glioblastoma [39]. Consistent with previous studies, we also demonstrated that the levels of CHI3L1 were higher in tissues and serum from GC patients, and high expression of CHI3L1 in GC was associated with shorter OS. Notably, we further explored the diagnosis of CHI3L1 as the serum biomarker for GC. We found that CHI3L1 exhibited an outstanding diagnostic value with a superior AUC value. However, no correlation was observed between CHI3L1 and immune cells based on TCGA database. TFF2 (the trefoil factor 2) [40], as a member of trefoil factor, has demonstrated potential importance in the diagnosis and prognosis prediction of GC. Although there were no effects of TFF2 silencing on GC cells, the role of TFF2 as the serum biomarker was demonstrated in our study, and further experiments needed to be conducted. Importantly, our study found that the inconsistency of TFF2 expression between serum and tissues might have resulted from the different infiltration of B cell memory. In this model, low expression of FCGBP (IgGFc‐binding protein) [41] and VSIG2 (V‐set and Ig domain‐containing protein 2) [42, 43] was associated with tumor progression, but their roles and mechanisms in GC remain unclear. FCGBP is initially detected in the intestinal epithelium, playing a vital role in innate mucosal epithelial defense, metastasis, and tumor immunity. In our study, we first identified its lower expression in GC patients compared with the normal tissues, and the results of cell viability assays revealed that it had an inhibitory effect on growth of GC cell line. Additionally, it seemed that GC patients with low expression of FCGBP had a shorter survival time and their GC tissues were infiltrated with more B cell memory. There are only a few studies about the expression and role of VSIG2, and it has not been reported in GC. The latest findings suggested that VSIG2 functioned as a key molecule in tumor and immune cells [44]. Based on TCGA database, we found that lower expression of VSIG2 was observed in GC patients with higher Tregs infiltration. Importantly, the level of VSIG2 could also be detected in serum and predicted a better diagnostic value for GC patients. Cell growth was accelerated after silencing VSIG2, suggesting a tumor suppressor role of VSIG2 in GC.

Furthermore, this study uncovered significantly different immune infiltration patterns between the CHI3L1^low^ + FCGBP^high^ + VISIG2^high^ + TFF2^high^ and CHI3L1^high^ + FCGBP^low^ + VISIG2^low^ + TFF2^low^ groups. This revealed that the CHI3L1^high^ + FCGBP^low^ + VISIG2^low^ + TFF2^low^ group was significantly correlated with B cell memory and Tfh. B cell memory infiltration was observed in GC and associated with tumor progression and prognosis, suggesting it could serve as an independent protective prognostic factor [45, 46]. Tfh cells, also called T‐cell follicular helper cells, specially provide cytokines to promote B cell development [47]. Tregs, a subset of T lymphocytes, were found to be massively infiltrated into GC tissues, indicating their pivotal role in GC development and progression [48]. Tracking T cell clonotypes in different tissue types, Tfh and Treg cells were enriched in viable cancer cells, emphasizing their mutual regulatory effect in cancer [49, 50]. Nevertheless, the relationships between the levels of FCGBP, VSIG2, and TFF2 and the infiltration of B cell memory and Tregs remained unknown. In our study, the results of FCM analysis revealed inconsistent expressions of FCGBP, VSIG2, and TFF2 between GC tissues and serum, resulting from lower B cell memory and Tregs infiltration in peripheral blood. This study also assessed a negative correlation between B cell memory and FCGBP and VSIG2 levels, as well as Tregs and TFF2 levels, which is a novel finding of this study. Additionally, a correlation was observed between the Type I IFN response and the CHI3L1^high^ + FCGBP^low^ + VISIG2^low^ + TFF2^low^ group. Type I IFNs are known for their key role in anticancer immune responses by acting upon tumors and immune cells [51, 52]. However, the specific mechanisms linking B cell memory and Tfh to the Type I IFN response in GC have not been demonstrated.

In summary, this study reports, for the first time, four promising biomarkers that can independently diagnose and predict GC and OS of patients with GC. A prognostic nomogram based on clinical information, including age and stage, was constructed, which performed well in predicting the prognosis of patients with GC. The results of this study confirm that these four promising biomarkers could serve as powerful and novel candidate biomarkers for GC and be valuable clinical tools for GC diagnostic and prognostic management. Despite the significance of this study, further efforts are still needed to validate these insights through experiments and clinical trials.

## Author Contributions

Yi Liu, Bingxian Bian, Lisong Shen, and Hui Chen conceptualized and designed the study plan; Yi Liu, Bingxian Bian, and Shiyu Chen performed the analysis and produced the Figures; Yi Liu, Bingxian Bian, Lisong Shen, and Hui Chen wrote and revised the manuscript; Shiyu Chen, Bingqian Zhou, and Peng Zhang assisted in analyzing and manuscript writing. All authors have read and agreed to the published version of the manuscript.

## Ethics Statement

All clinical samples used in this study were approved by the Institutional Review Board and Ethics Committee of Xinhua Hospital.

## Conflicts of Interest

The authors declare no conflicts of interest.

## Supporting information


Data S1

## Data Availability

The publicly available data from TCGA and GEO databases in this study is analyzed. All analyses were performed using the R version 3.6.0. We performed clustering, pathway, and survival analysis by using R/Bioconductor tools.
