# Supplementary material for: Identification and Validation of Four Serum Biomarkers With Optimal Diagnostic and Prognostic Potential for Gastric Cancer Based on Machine Learning Algorithms
Source: Cancer Med. 2025 Mar 14;14(6):e70659. doi: 10.1002/cam4.70659 (PMC11907202; doi:10.1002/cam4.70659)
Supplement: Supplementary file 1 — Data S1 [file CAM4-14-e70659-s001.docx]

**Supplementary Information**

**Identification and validation of four serum biomarkers with optimal diagnostic and prognostic potential for gastric cancer based on machine learning algorithms**

1. **Figure S1- S9.**
2. **Table S1- S4.**

**Figure S1-S9**

**
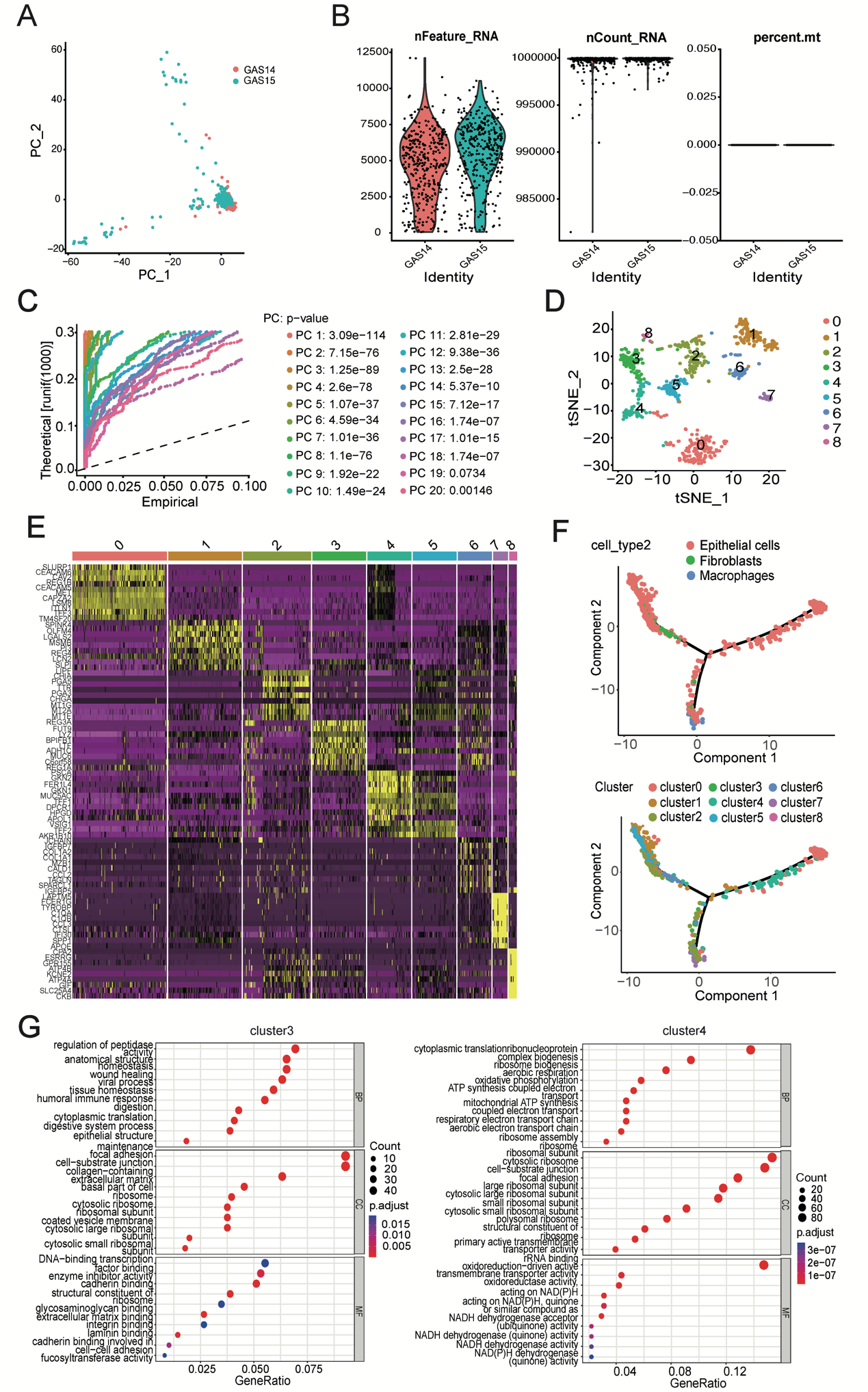
**

**Figure S1.** sc-RNAseq analysis of stem-related genes in GSE112302 dataset. The PCA (A) and QC (B) were performed to reduce dimensionality of the scRNA-seq data by employing R package. (C) The top 20 PCs with significant differences were identified at P < 0.05. (D-E) The t-SNE algorithm was used to aggregate the cell clusters, and the 10% of differential marker genes were displayed with heatmap. (F) Pseudo-time and trajectory analyses were performed to indicate the distribution of cell clusters. (G) Enrichment analysis of marker genes for cluster 3 and 4.


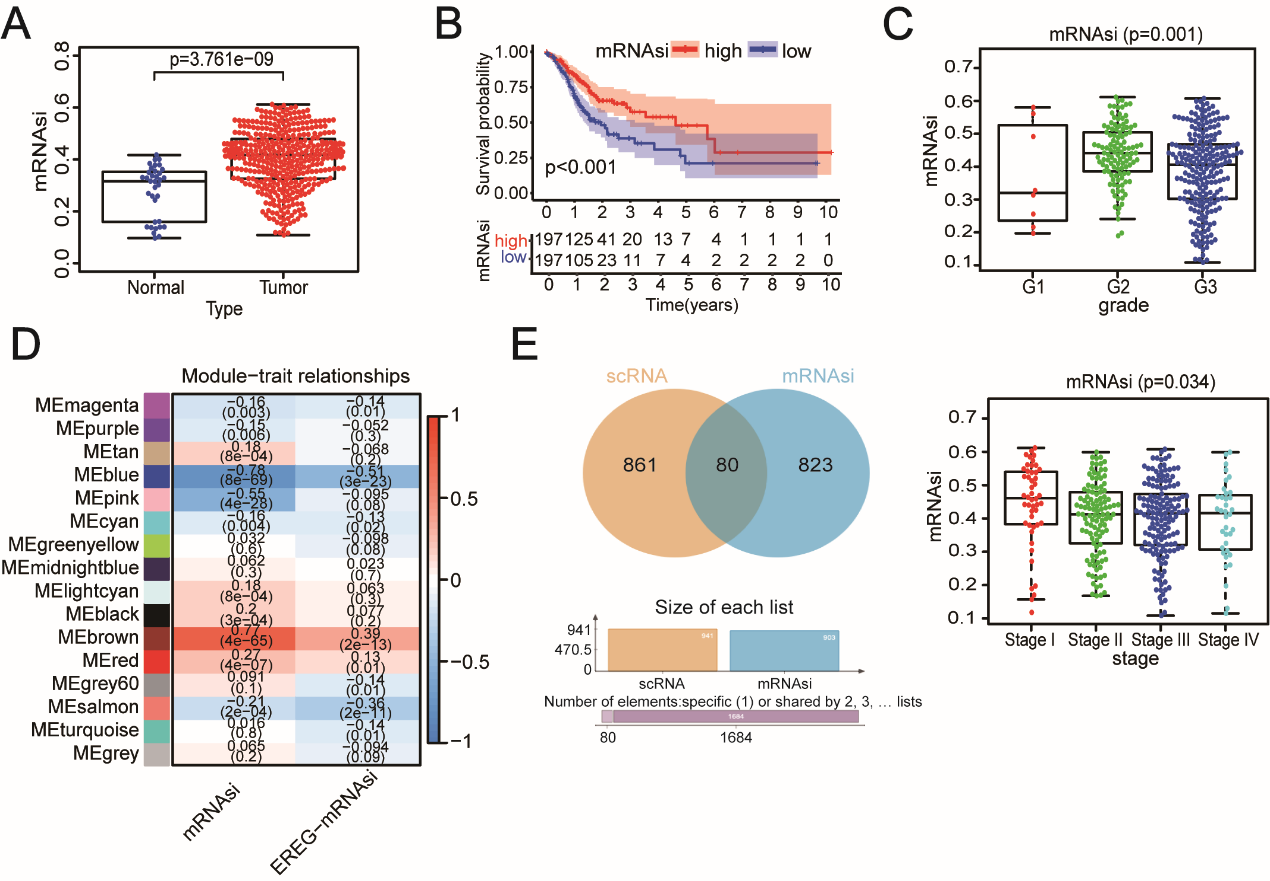


**Figure S2.** mRNAsi analysis of stem-related genes in TCGA database. (A) mRNAsi in GC and normal tissues. (B) Kaplan-Meier analysis of the OS between high-risk and low-risk group. (C) Comparison of mRNAsi in different grades and stages. (D) Correlation between the gene module and mRNAsi, and correlation coefficient increased in size from blue and red. (E) The intersection of the analytic results of scRNA-seq and mRNAsi shown by Venn diagram.


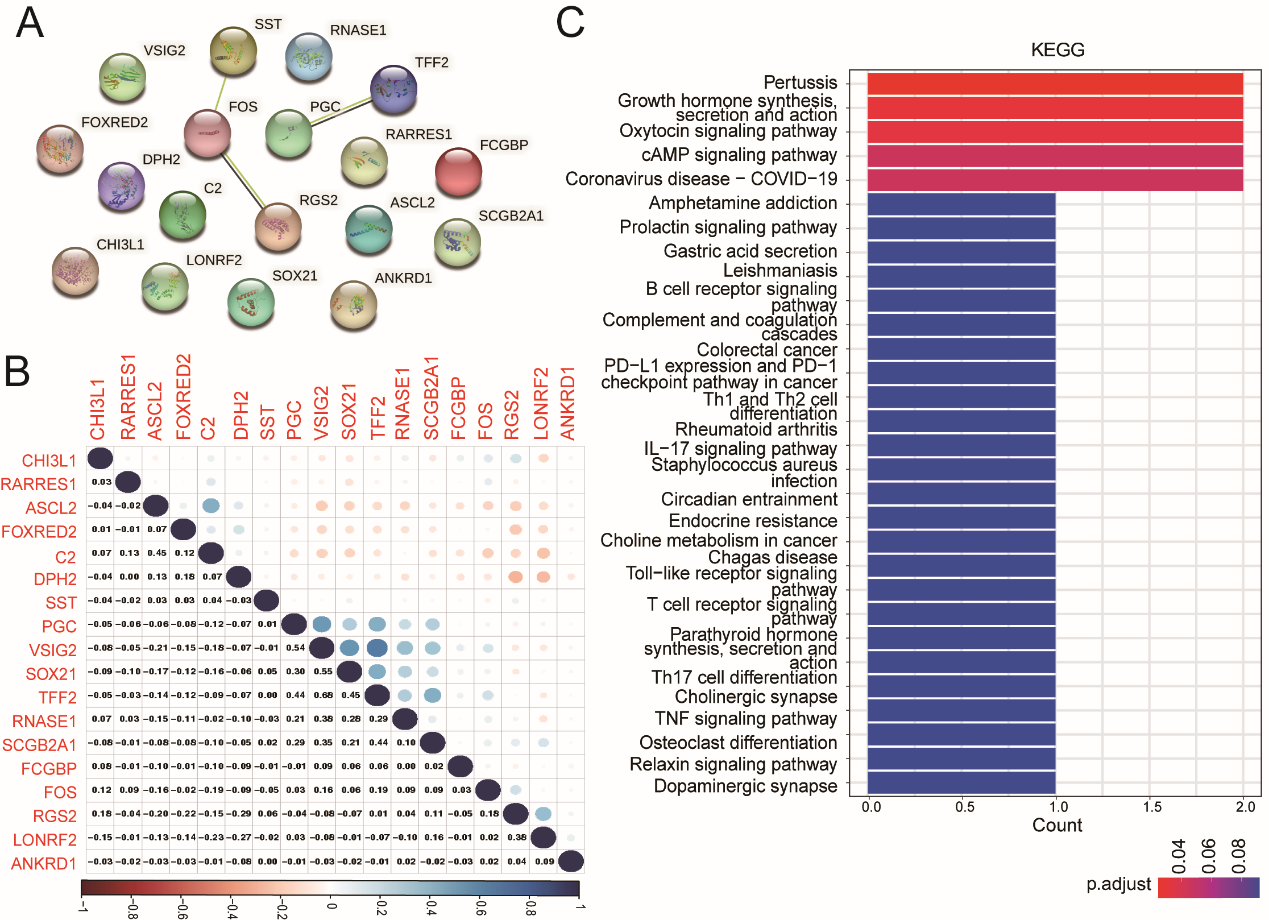


**Figure S3.** PPI network construction and functional enrichment analysis of the hub genes. (A)The STRING analysis of the relationship among hub genes. (B) The mutual correlation among hub genes. (C) The KEGG analysis of the involvement of cell signaling pathways.


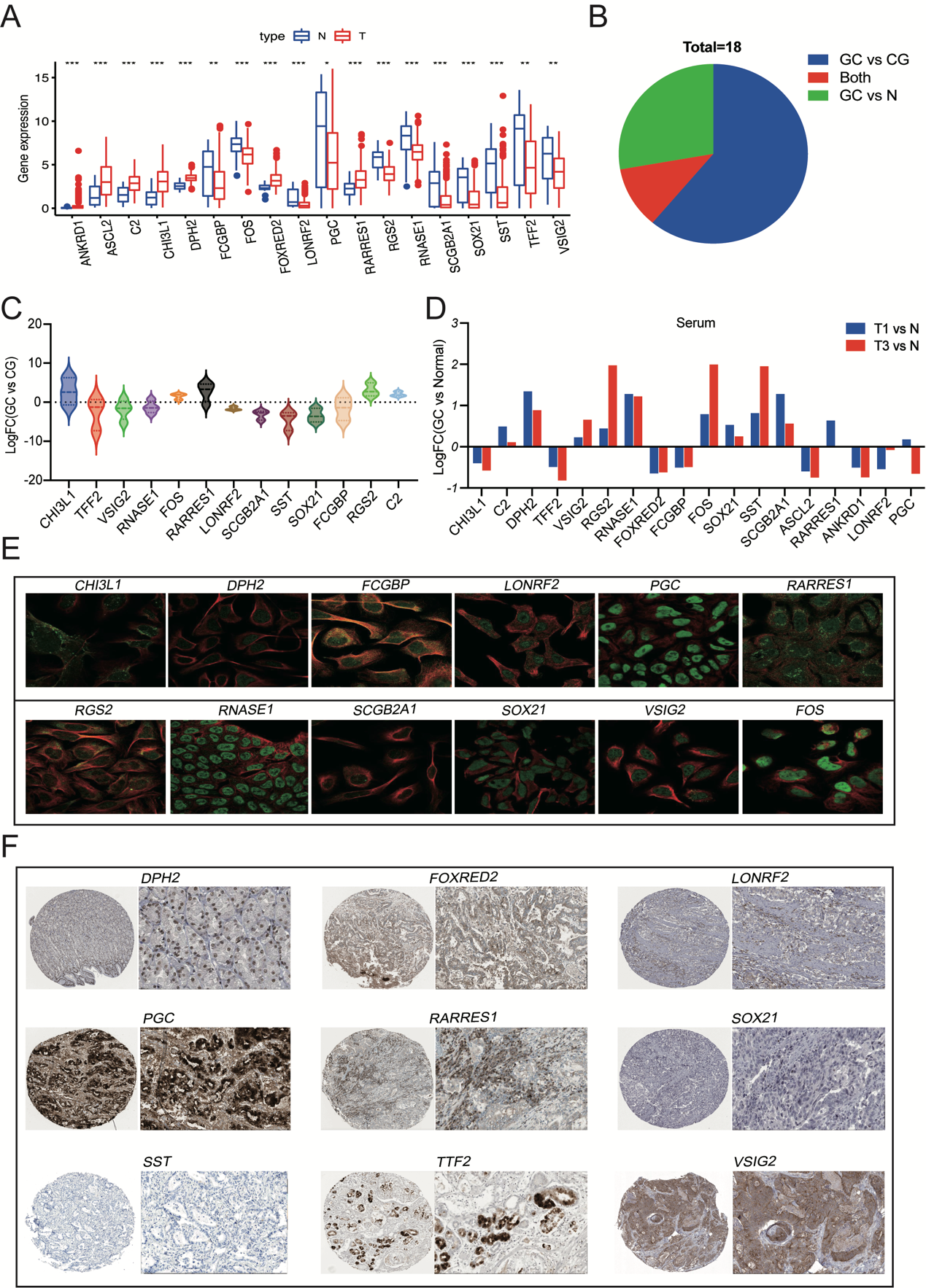


**Figure S4.** Expression and cellular localization of hub genes. (A) The mRNA expression levels of the 18 hub genes in GC obtained from TCGA-STAD. (B) The percentage of the DEGs from three groups within the 18 hub genes. (C) Boxplot showed the DEGs between GC and CG. (D) The mRNA expression of hub genes in serum. (E and F) Immunofluorescence and Immunohistochemistry analyses of proteins encoded by the hub genes in cells and GC tissues from HPA databases. A549 cell was used to determine the expression and localization of LONTF2. The PCG level was detected in CACO2 cell. The levels of RARRES1 and RANSE1 were shown in RT-4 cell. AF22 cell was used to detect the expression and localization of SOX21. Other proteins encoded by hub genes were detected in U2OS cell. Statistically significant values are indicated by asterisks (* p < 0.05, ** p < 0.01, *** p < 0.001).


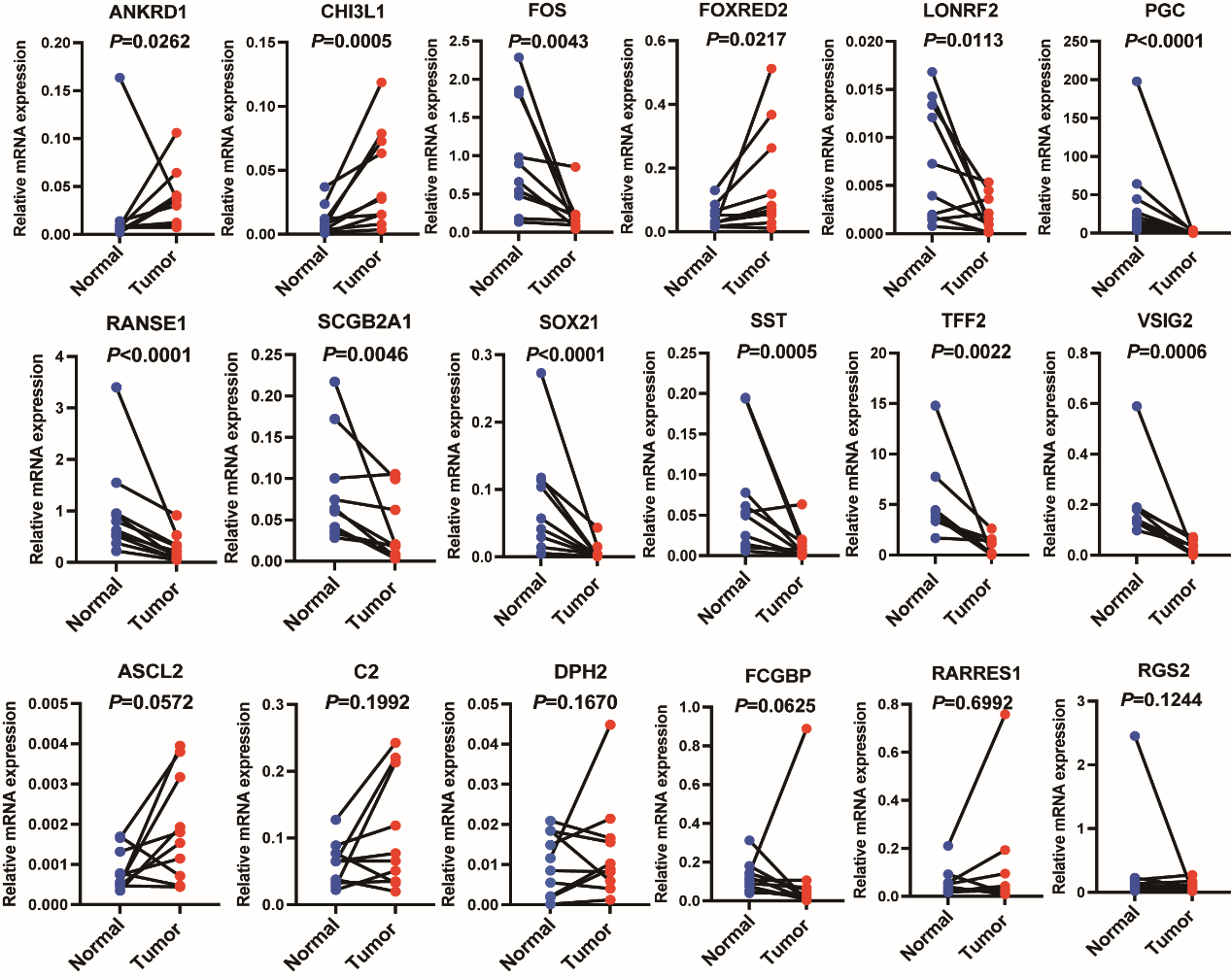


**Figure S5.** Relative mRNA expression of the hub genes in GC tissues using qRT-PCR.


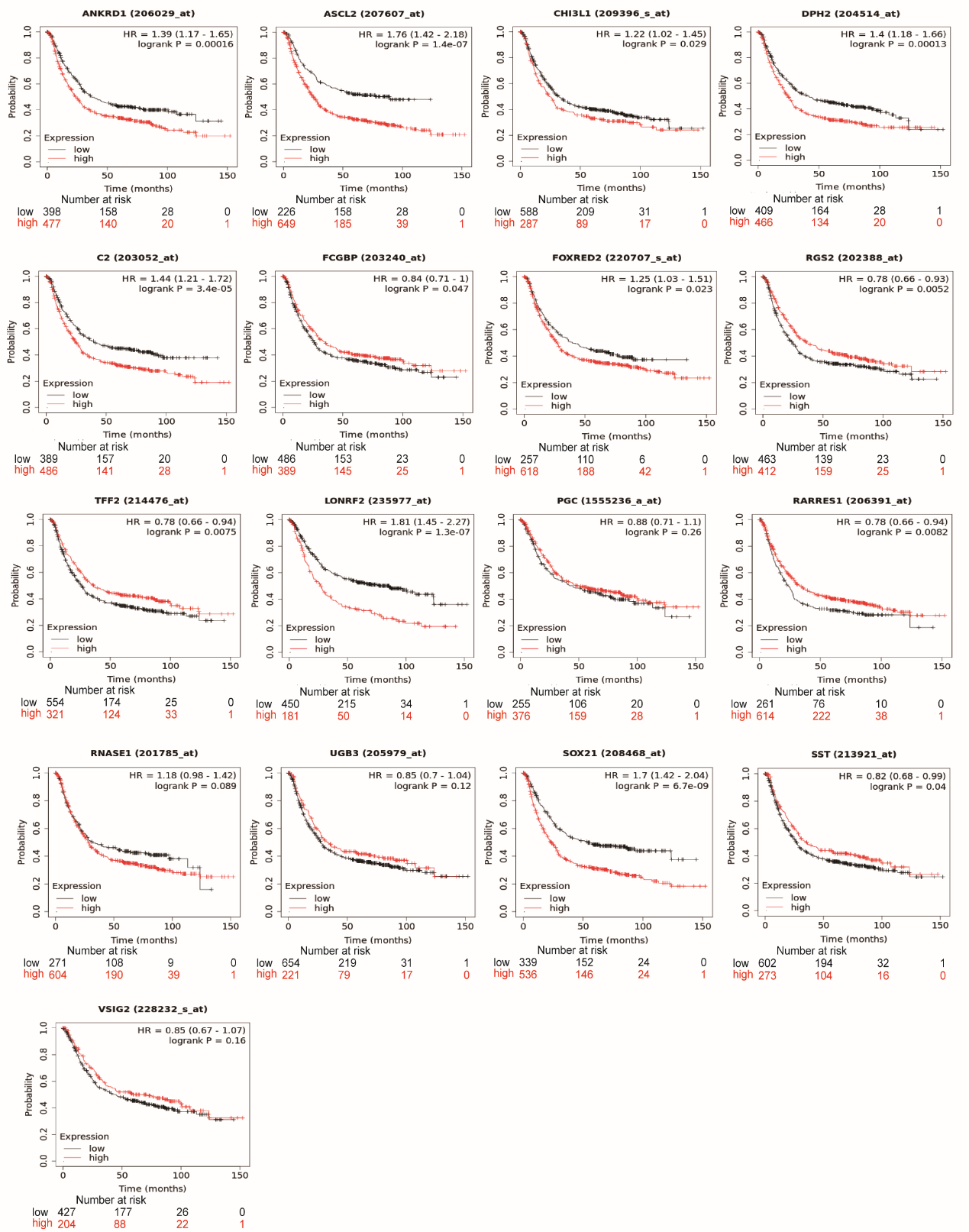


**Figure S6.** Kaplan-Meier analysis of the hub genes based on clinicopathological characteristics of GC patients from GEO database.


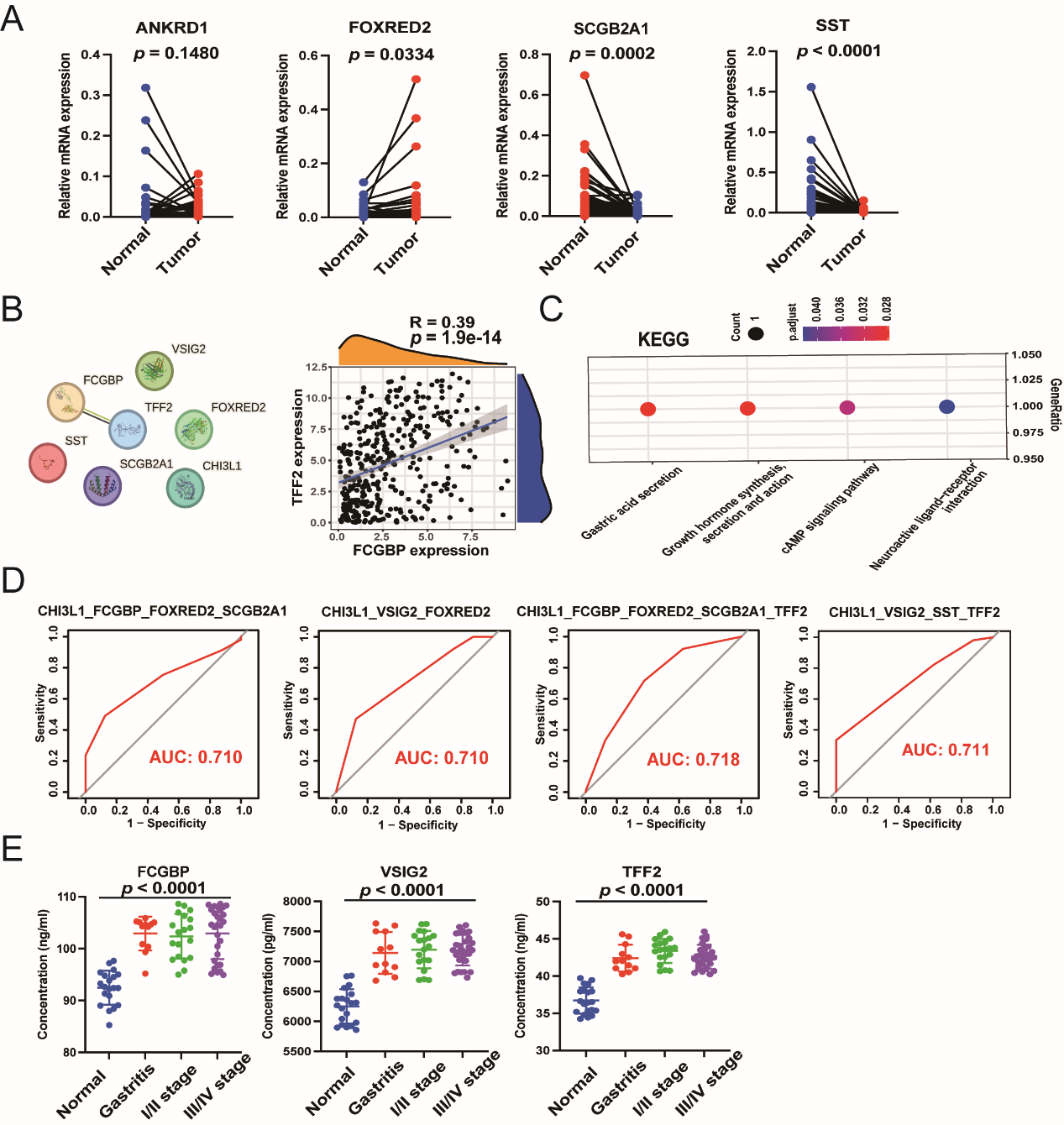


**Figure S7.** Experimental and bioinformatics analysis of 4 promising biomarkers in serum of GC. (A) qRT-PCR analysis of ANKRD1, FOXRED2, SCGB2A1 and SST mRNA levels in 54 pairs of normal and GC samples. (B) The STRING analysis of relationships between 7 genes. (C) The KEGG analysis of the involvement of cell signaling pathways of 7 genes. (D) The top 4 panels with much higher AUC values were listed by ROC analysis. (E) The Elisa analysis of FCGBP, VSIG2 and TFF2 levels in serum of 20 normal samples, 12 gastritis patients, 19 I/II stage of GC patients and 29 III/IV stage of GC patients. Statistically significant *p* values are indicated.


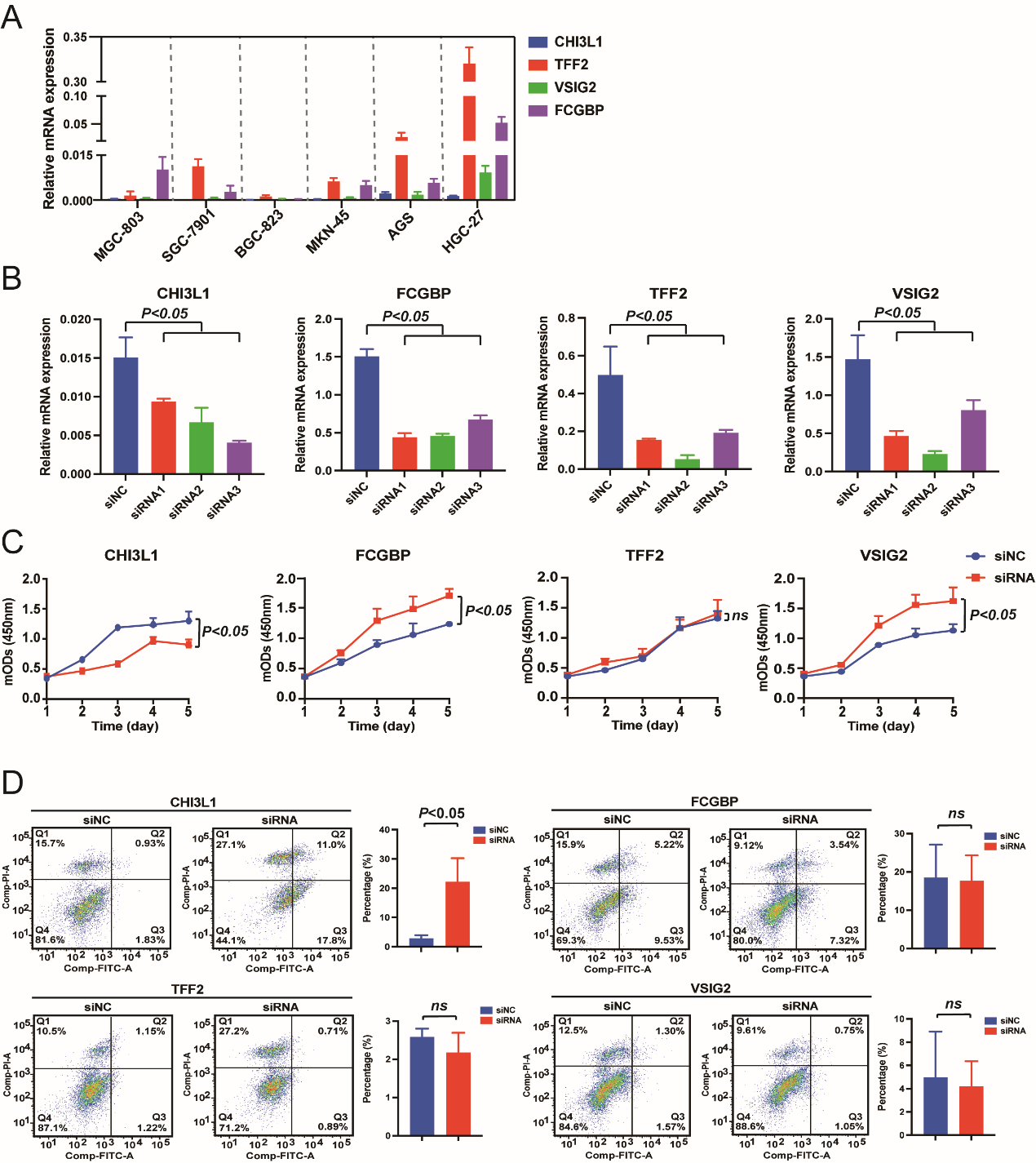


**Figure S8.** The effects of 4 biomarkers on cell viability of HGC-27 cell line. (A) Analyze relative mRNA levels of the 4 biomarkers in 6 kinds of GC cell lines using qRT-PCR. (B) qRT-PCR analysis of the silencing effects of the different siRNAs on mRNA levels of 4 biomarkers in HGC-27 cell line. (C) CCK8 analysis of the cell proliferation ability between siNC and siRNA groups. (D) FCM analysis of the cell apoptosis between siNC and siRNA groups. Statistically significant *p* values are indicated, and *ns* represents no significant difference.


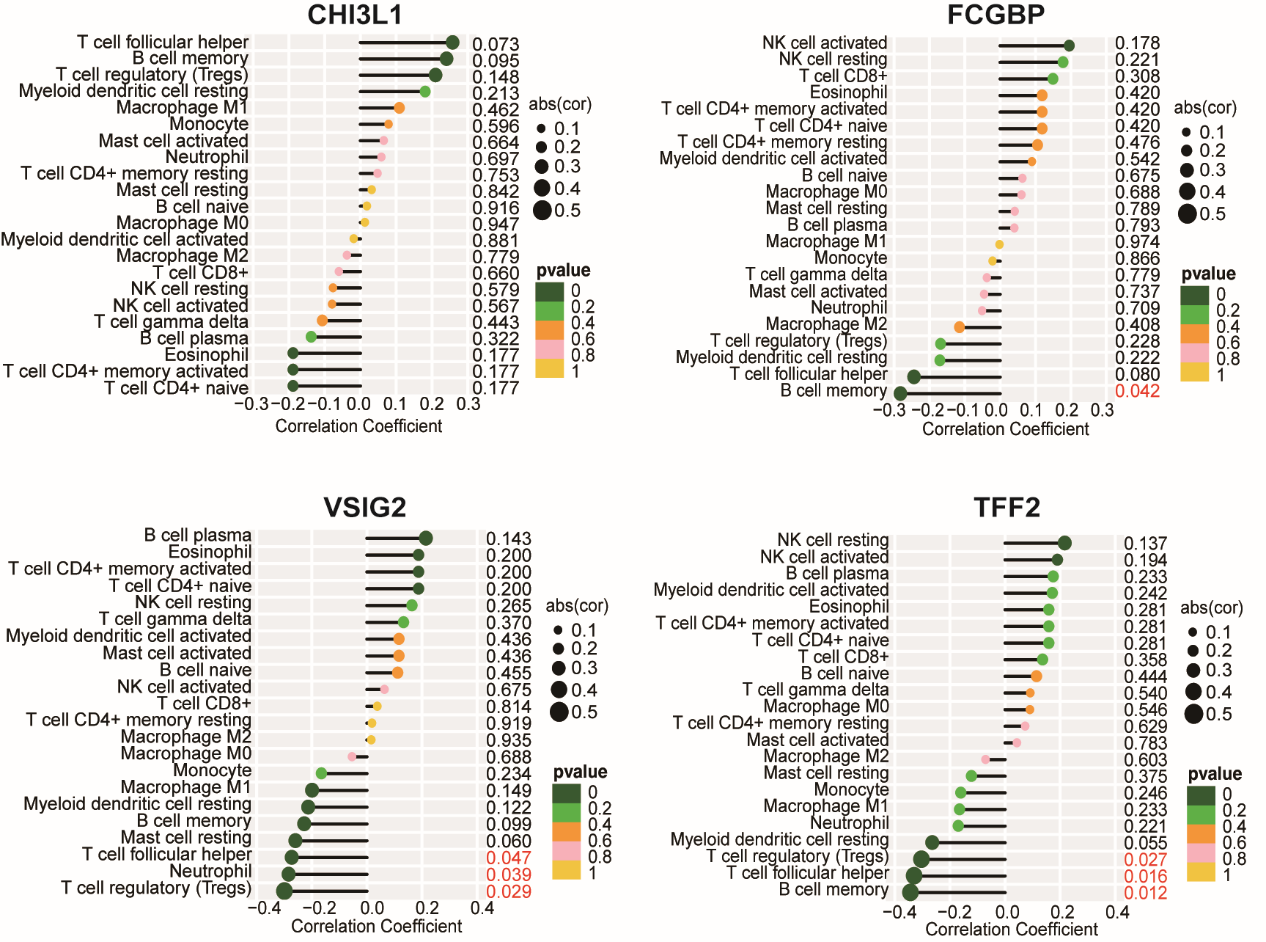


**Figure S9.** The relationships between 4 promising biomarkers and immune cells.

**Table S1 - S4**

Table S1. The primers used in the study

| **Name** | **Forward sequence (5’-3’)** | **Reverse sequence (5’-3’)** |
| --- | --- | --- |
| ACTIN | TGAAGTACCCCATCGAGCACGGCA | GATAGCACAGCCTGGATAGCAACG |
| PGC | GTCGTCCACCTACTCCACCAATG | ATTCTCACTCAAGCCGAACTCCTG |
| LONRF2 | GATGGCTATAACACAGCGGACATTG | ATCGTGGAGAGCGGCAAGTTC |
| ANKRD1 | CGCCGATGGATCTGGTGCTAC | GCTATGCGAGAGGTCTTGTAGGAG |
| RARRES1 | TTCAGGAAGGTGAGGGACGTTTG | CATTGATGGTTGGTCTGGGTTTCTG |
| ASCL2 | GCGTGAAGCTGGTGAACTTGG | CGGATGTACTCCACGGCTGAG |
| SCGB2A1 | ACTCCTGGAGGACATGGTTGAAAAG | AGCGGCATCACTGTCTATGAACTC |
| SOX21 | TGAACGCCTTCATGGTGTGGTC | GCCGCTTCTCCGACTCTGTG |
| FOS | CCAAGCGGAGACAGACCAACTAG | GTCGGTGAGCTGCCAGGATG |
| FCGBP | CTGGATGGGCACCGATTCAATTTC | TGGCTACAGTGACAGTGAAGTTCTC |
| FOXRED2 | AATGTGCTGATCCTGGGTCGTG | ATTGTTGATGGCTCTGAGGTCTCC |
| RANSE1 | TGCTTGTCCTGATACTGCTGGTG | ATATGCTGCCGCTGGAATTTCTTG |
| RGS2 | AGTGCTATGTTCTTGGCTGTTCAAC | CTCGCTTCTCCTCGCTCTTGTG |
| VSIG2 | TGGTTCTAAGTCAAAGCGGGTCAG | CTTGGCAGAGGTAGGTTCCAGTATC |
| TFF2 | AGACCGAAGAAACTGTGGCTACC | GCACCAGGGCACTTCAAAGATG |
| DPH2 | GTGTACGAGCTGGAGCGAGTC | ACAGCCACAGCATCTCCCAATAG |
| C2 | GCCACCAATCCCACCCAGAAG | ACAGTCCAGGAGCAGGTAGAGG |
| CHI3L1 | AGTGGAATGATGTGACGCTCTACG | CTGGGTGTTGGAGGCTATCTTGG |
| SST | CTGCGCTGTCCATCGTCCTG | GGCATCATTCTCCGTCTGGTTGG |

Table S2. Summary of patient characteristics (GC tissues)

| **Clinicopathological feature** | **Number of cases** |
| --- | --- |
| **Gender** |  |
| Male | 46 |
| Female | 18 |
| **Age(years)** |  |
| ≥60 | 50 |
| <60 | 14 |
| **Lymphatic invasion** |  |
| Yes | 38 |
| No | 26 |
| **TNM stage** |  |
| І/Ⅱ | 27 |
| Ⅲ/Ⅳ | 37 |

Table S3. Summary of patient characteristics (Serum)

| **Clinicopathological feature** | **Number of cases** |
| --- | --- |
| **Gender** |  |
| Male | 37 |
| Female | 11 |
| **Age(years)** |  |
| ≥60 | 35 |
| <60 | 13 |
| **Lymphatic invasion** |  |
| Yes | 10 |
| No | 13 |
| Missing | 25 |
| **TNM stage** |  |
| І/Ⅱ | 19 |
| Ⅲ/Ⅳ | 29 |

Table S4. Summary of patient characteristics (Serum)

| **Clinicopathological feature** | **Number of Gastritis patients** | **Number of І/Ⅱ stage GC** | **Number of Ⅲ/Ⅳstage GC** |
| --- | --- | --- | --- |
| **Gender** |  |  |  |
| Male | 8 | 15 | 22 |
| Female | 4 | 4 | 7 |
| **Age(years)** |  |  |  |
| ≥60 | 6 | 14 | 17 |
| <60 | 6 | 5 | 12 |
| **Expressions of Tumor markers**  **(mean ± SEM)** |  |  |  |
| CHI3L1 (pg/ml) | 415.2 ± 15.70 | 412.9 ± 15.33 | 400.8 ± 14.00 |
| FCGBP (ng/ml) | 102.9 ± 3.252 | 102.3 ± 4.303 | 102.9 ± 4.924 |
| VSIG2 (pg/ml) | 7141 ± 348.7 | 7195 ± 311.1 | 7185 ± 256.0 |
| TFF2 (ng/ml) | 42.41 ± 1.802 | 43.36 ± 1.600 | 42.59 ± 1.589 |
| AFP (ng/ml) | 2.823 ± 1.547 | 7.435 ± 19.32 | 127.8 ± 634.1 |
| CEA (ng/ml) | 1.578 ± 0.912 | 2.275 ± 1.970 | 4.850 ± 7.127 |
| CA199 (U/ml) | 9.353 ± 6.048 | 553.9 ± 2309 | 540.3 ± 2224 |
| CA724 (U/ml) | 4.987 ± 5.194 | 27.47 ± 104.4 | 30.13 ± 81.02 |
| CA125 (U/ml) | 13.81 ± 9.791 | 23.38 ± 51.41 | 28.78 ± 42.89 |
| CYFRA211 (ng/ml) | 2.768 ± 1.853 | 2.659 ± 2.211 | 5.723 ± 8.587 |
| CA153 (U/ml) | 11.96 ± 6.521 | 11.85 ± 16.22 | 10.56 ± 4.196 |
| CA242 (IU/ml) | 3.213 ± 2.239 | 14.50 ± 46.37 | 15.60 ± 41.44 |
| NSE (ug/l) | 14.76 ± 5.513 | 13.39 ± 2.888 | 12.64 ± 2.835 |
| SCC (ng/ml | 0.843 ± 0.288 | 0.762 ± 0.342 | 1.083 ± 1.248 |
| CA50 (U/ml) | 5.314 ± 3.744 | 36.07 ± 123.7 | 40.54 ± 132.3 |
